# Multimodal Multitask Learning for Predicting Depression Severity and Suicide Risk Using Pretrained Audio and Text Embeddings: Methodology Development and Application

**DOI:** 10.2196/66907

**Published:** 2025-10-30

**Authors:** Ya-Han Hu, Ruei-Yan Wu, Min-Yi Su, I-Li Lin, Cheng-Che Shen

**Affiliations:** 1 Department of Information Management, National Central University, No. 300, Zhongda Rd., Zhongli Dist., Taoyuan City, Taiwan; 2 Asian Institute for Impact Measurement and Management, National Central University, Taoyuan City, Taiwan; 3 Graduate School of Resources Management and Decision Science, Management College, National Defense University, Taipei City, Taiwan; 4 Department of Radiology, Ditmanson Medical Foundation Chia-Yi Christian Hospital, Chiayi, Taiwan; 5 Jianan Psychiatric Center, No. 539, Yuzhong Rd., Rende Dist., Tainan City, 71742, Taiwan, 886 62795019 ext 1537; 6 School of Medicine, National Yang Ming Chiao Tung University, Taipei, Taiwan

**Keywords:** depression severity, suicide risk, multitask learning, multimodal learning, transfer learning, mental health, mental illnesses, mental disorders, depression, depressed, major depressive disorder, MDD, depressive disorder, machine learning, ML, artificial intelligence, AI, algorithms, predictive models, predictive analytics, deep learning, early detection

## Abstract

**Background:**

Depression is a critical psychological disorder necessitating urgent assessment and treatment, given its strong association with increased suicide risk (SR). Effective management hinges on promptly identifying individuals with high depression severity (DS) and SR. While machine learning and deep learning have advanced the identification of DS and SR, research focusing on both aspects simultaneously remains limited and requires further refinement.

**Objective:**

This study aimed to evaluate whether our proposed methods, which integrate multitask learning (MTL), multimodal learning, and transfer learning, enhance the efficacy of deep learning models in the joint classification of DS and SR.

**Methods:**

This study proposed a multitask framework employing a multimodal fusion strategy for pretrained audio and text embeddings to concurrently assess DS and SR. Data encompassing Chinese audio recordings and clinical questionnaire scores from 100 patients with depression and 100 healthy controls were used. Preprocessed audio and text data were transformed into pretrained embeddings and integrated using concatenation and hard parameter sharing. Single-task learning (STL) models (DS and SR tasks) were evaluated with different embeddings and further compared with the MTL models.

**Results:**

The STL models demonstrated exceptional DS prediction (area under the curve [AUC]=0.878) using wav2vec 2.0 combined with ERNIE-health, and SR prediction (AUC=0.876) using HuBERT combined with ERNIE-health. The MTL models significantly improved SR prediction over DS prediction, achieving the highest DS classification (AUC=0.887) with wav2vec 2.0 combined with ERNIE-health, and SR classification (AUC=0.883) with HuBERT combined with ERNIE-health.

**Conclusions:**

The findings of this study underscore the effectiveness of the proposed MTL models using specific pretrained audio and text embeddings in enhancing model performance. However, we advocate for cautious implementation of MTL to mitigate potential negative transfer effects. Our research presents a method that is both promising and effective, offering an objective approach for accurate clinical decision support in the parallel diagnosis of DS and SR.

## Introduction

### Background

In its pervasive embrace, depression, an ever-expanding mental malady, reaches across the globe, leaving its mark on approximately 280 million lives [1]. Neglecting proper care of patients with depression can lead to dire consequences, as research has shown that individuals with this condition face a staggering 20-fold higher risk of suicide than the general population [2,3], exposing a troubling link between depression and suicide [4-6].

In clinical practice, regular and comprehensive assessments of depression severity (DS) and suicide risk (SR) remain challenging due to time and resource constraints [7,8]. Traditional evaluations often rely on subjective and infrequent self-reports from patients or caregivers, which are susceptible to recall bias, cognitive limitations, and social stigma [9,10]. These issues are particularly acute in high-volume settings, where limited consultation time may hinder the timely identification of critical warning signs related to mental health deterioration or suicidal ideation.

Machine learning has demonstrated strong potential in predicting DS and SR, using text and audio data (eg, [11,12]). Text-based approaches have extracted clinically meaningful insights from medical narratives [13,14], while speech analysis has improved predictive accuracy by identifying vocal biomarkers linked to depression and suicide, such as reduced intensity, slower tempo, and increased hesitation [15-17]. These advances have driven the development of multimodal learning (MML) frameworks for mental health prediction. Although effective in detecting depression [18,19], applications of MML to SR prediction remain limited—likely due to the scarcity of high-quality annotated data in this sensitive domain [20,21].

Given the frequent co-occurrence of depression and suicide in clinical populations [22-24], SR prediction is inherently linked to depression assessment. This conceptual interdependence highlights the potential of multitask learning (MTL) for simultaneously modeling related mental health outcomes. Benton et al [25] demonstrated the utility of MTL by jointly predicting SR and other psychiatric conditions using social media data. With the rapid advancement of deep learning, transfer learning (TL) has also emerged as a promising strategy to address data scarcity, with recent studies showing that fine-tuning pretrained models on downstream mental health tasks can significantly enhance predictive performance (eg, [26,27]).

However, the current literature reveals several gaps. First, data source diversity remains limited, with most studies relying on datasets from English-speaking populations (eg, [28,29]). In addition, many analyses are based on social media platforms (eg, [25,30]) or public datasets (eg, [27,31]), which often lack clinical relevance. Second, most SR prediction studies have underutilized TL for audio processing, despite its successful application in related domains such as speech emotion recognition [32,33]. Third, although the comorbidity and shared clinical features of DS and SR are well documented [22-24], few studies have applied MTL to model these outcomes jointly.

While recent advances in MML and MTL have shown promise in mental health prediction, few studies have jointly modeled DS and SR using clinically grounded, non-English data. Furthermore, the potential of TL to improve model generalizability across tasks and modalities remains underexplored in Chinese-language clinical contexts. These gaps motivate this study’s unified framework, which integrates MML, MTL, and TL to support scalable and efficient mental health screening in real-world clinical settings for Chinese-speaking populations.

The key contributions of this work are three-fold: (1) the development of the first integrated framework that combines MML, MTL, and TL for the joint prediction of DS and SR in Chinese contexts; (2) empirical validation of MML approaches compared to single modality baselines in a non-English clinical setting; and (3) demonstration of the effectiveness of MTL in modeling related mental health constructs. By addressing linguistic, cultural, and resource-specific challenges, this framework supports scalable and efficient screening in high-volume clinical environments, addressing an urgent need in early mental health assessments and targeted interventions.

### Related Work

Research on predictive models in mental health has traditionally adopted single-task approaches, predicting either depression or suicide independently [34-38]. These studies have primarily relied on text, audio, or other features, such as structured electronic health records [39] and social media images [40], to build predictive models. Recent advancements in text processing technologies have facilitated a shift from conventional hand-crafted features toward sophisticated automated feature learning approaches, exemplified by the heterogeneous graph convolutional network of Wang et al [14]. Concurrently, speech-based analyses have gained prominence for their capacity to capture nuanced vocal markers indicative of mental health conditions [11,15].

MTL has emerged as a promising framework for mental health assessment, as summarized in Table 1, aligning with the clinical observation that psychiatric conditions often co-occur and share common underlying mechanisms [22-24]. By jointly learning related tasks, MTL facilitates representation sharing and information transfer, thereby mitigating data sparsity and overfitting issues [41-43]. Benton et al [25] pioneered the use of deep neural networks to simultaneously predict depression and SR using Twitter data.

**Table 1.** Summary of key literature on multitask learning for depression severity and suicide risk prediction.

Study	Dataset	Language	Sample	Modality		TL^a^	Task		Method
				A^b^	T^c^		DS^d^	SR^e^	
Benton et al [25]	Multiple Twitter datasets	English	9611 users	No	Yes	No	Yes	Yes	DNN^f^
Qureshi et al [31]	DAIC-WOZ^g^	English	189 recordings	Yes	Yes	No	Yes	No	LSTM^h^
Ophir et al [30]	Facebook posts	English	83,292 postings	No	Yes	No	No	Yes	ANN^i^
Qureshi et al [28]	DAIC-WOZ, CMU-MOSEI^j^	English	189 recordings	No	Yes	Yes	Yes	No	LSTM
Dumpala et al [44]	FORBOW^k^	English	526 recordings	Yes	No	Yes	Yes	No	CNN^l^
Yang et al [45]	Chinese micro-blog	Chinese	6100 comments	No	Yes	Yes	Yes	No	DNN
Ghosh et al [46]	CEASE	English	2539 sentences	No	Yes	Yes	Yes	No	Bi-GRU^m^
Buddhitha and Inkpen [29]	CLPsych 2015 Twitter, UMD^n^, SMHD^o^	English	—^p^	No	Yes	No	No	Yes	CNN
Teng et al [26]	AVEC^q^ 2019 DDS Challenge Dataset, CMU-MOSEI	English	23,454 video clips and 275 users	Yes	Yes	Yes	Yes	No	DNN
Yang et al [27]	CEASE	English	2393 sentences	No	Yes	Yes	No	Yes	BERT^r^
This study	Self-collected	Chinese	200 users	Yes	Yes	Yes	Yes	Yes	DNN

a TL: transfer learning.

b A: audio modality.

c T: text modality.

d DS: depression severity.

e SR: suicide risk.

f DNN: deep neural network.

g DAIC-WOZ: distress analysis interview corpus-Wizard of Oz.

h LSTM: long short-term memory.

i ANN: artificial neural network.

j CMU-MOSEI: CMU multimodal opinion sentiment and emotion intensity.

k FORBOW: families overcoming risks and building opportunities for wellbeing.

l CNN: convolutional neural network.

m Bi-GRU: bidirectional GRU.

n UMD: University of Maryland Reddit suicidality dataset.

o SMHD: self-reported mental health diagnoses dataset.

p Not applicable.

q AVEC: audio/visual emotion challenge.

r BERT: bidirectional encoder representations from transformers.

Several studies listed in Table 1 have incorporated MML to improve predictive performance. By integrating diverse data types, MML leverages complementary information to enable a more comprehensive characterization of mental states. Qureshi et al [31], for example, demonstrated enhanced depression prediction accuracy using long short-term memory models trained on combined textual and acoustic features from the DAIC-WOZ (distress analysis interview corpus-Wizard of Oz) dataset. Additionally, TL has also been increasingly adopted in these frameworks to address the challenge of limited labeled data. Teng et al [26] applied depression detection with sentiment assistance through deep neural networks and TL techniques on the AVEC (audio/visual emotion challenge) 2019 DDS Challenge and CMU-MOSEI (CMU multimodal opinion sentiment and emotion intensity) datasets. Similarly, Yang et al [27] used MTL with a BERT-based model to incorporate time-perspective cues for suicidal ideation detection on the CEASE dataset.

Despite these advances, key limitations persist. First, most studies rely on English-language data. Furthermore, text-based models are often trained on social media content [25,30,45], while audio models rely on public datasets [26-29,31,44,46] that may lack relevance to real-world clinical scenarios, thereby potentially limiting their applicability. Second, most SR prediction models are still trained from scratch, with only a few studies (eg, [27]) leveraging TL to enhance model performance. Most critically, empirical research exploring MTL’s effectiveness for simultaneously predicting both DS and SR remains scarce. To our knowledge, only Benton et al [25] have conducted similar research, though their work was conducted exclusively in English on social media data.

To address these gaps, this study introduces a unified MML, MTL, and TL framework for the simultaneous prediction of DS and SR using Chinese-language data collected in clinical settings. This approach facilitates the development of culturally and linguistically tailored predictive models for Chinese-speaking populations. Moreover, by incorporating TL, the proposed framework retains knowledge acquired from source tasks, enabling efficient adaptation to downstream applications.

## Methods

### Ethical Considerations

This study received approval from the Institutional Review Board of Taichung Veterans General Hospital (approval number: SE21183B).

Every participant was required to complete and sign a participant consent form before their involvement. This form outlined the purpose and procedures of the study, potential risks and benefits, confidentiality measures, and voluntary participation rights. The completion of this form indicated their informed and voluntary consent to partake in the study. In the section of the participant consent form dedicated to “consent to participate,” participants were explicitly informed about the inclusion of a clause seeking their agreement to employ their personal data, information, or research outcomes for publication purposes. By completing and signing the participant consent form, participants signified their understanding and acceptance of the terms outlined, thereby granting their “consent for publication.” This agreement encompassed the use of their anonymized data and contributions in academic papers, reports, presentations, or other forms of scholarly dissemination.

### Study Population

We collected a Chinese chief complaint dataset, which includes data from 100 patients with depression from a regional hospital in southern Taiwan, along with 100 age- and sex-matched nondepressed counterparts selected at random, resulting in a total of 200 cases. To verify the matching process, we conducted tests. The chi-square test for gender in relation to the prevalence of the condition was not significant (*P*=.88). Similarly, the *t* test for age in relation to the prevalence of the condition was not significant (*P*=.60).

Each case in the dataset includes personal data, an audio recording describing the current situation, transcripts, and clinical questionnaire results. The audio recordings were acquired by instructing participants as follows: “Please take a minute to elucidate your recent emotions, life circumstances, and other states.” Subsequently, participants initiated the recording of their spoken expressions. Based on the questionnaire results, we conducted 2 specific clinical assessments: Hamilton Depression Rating Scale-17 (HAMD-17) [47] and SAD PERSONS scale [48]. DS was categorized into 3 levels: no depression (HAMD-17 score of 0‐7; sample size of 106), low/moderate depression (HAMD-17 score of 8-16/17-23; sample size of 21), and high depression (HAMD-17 score of ≥24; sample size of 73). SR was classified into 2 levels: low risk (SAD PERSONS score of 0‐3; sample size of 110) and moderate/high risk (SAD PERSONS score of 4-7/8-10; sample size of 90).

The demographic data for both groups can be found in Tables2  3. In the 3 DS groups, there were statistically significant differences between the 2 study groups regarding age (*P*=.048), educational level (*P*<.001), occupation (*P*=.01), and marriage (*P*=.001). In terms of educational level, the HAMD-17≤7 group exhibited higher levels compared to the 8<HAMD-17≤23 and HAMD-17≥24 groups, and the proportion of individuals employed was also higher in the HAMD-17≤7 group than in the 8<HAMD-17≤23 and HAMD-17≥24 groups (64/97, 66% vs 13/30, 43% and 33/73, 45%). In terms of marital status, the HAMD-17≤7 group had a higher proportion of married individuals and a lower proportion of divorced individuals. In the 2 SR groups, there were statistically significant differences between the 2 study groups regarding educational level (*P*<.001), occupation (*P*=.02), and marriage (*P*<.001). In terms of educational level, the SAD PERSONS≤3 group exhibited higher levels compared to the SAD PERSONS≥4 group. In terms of occupation, the proportion of individuals was also higher in the SAD PERSONS≤3 group than in the SAD PERSONS≥4 group (69/110, 62.7% vs 41/90, 45.6%). In terms of marital status, the SAD PERSONS≥4 group had a higher proportion of unmarried individuals.

**Table 2.** Demographic data of patients in the 3 depression severity groups.

Variable	HAMD-17^a^≤7 group (n=97)	8<HAMD-17≤23 group (n=30)	HAMD-17≥24 group (n=73)	*P* value
Sex, n (%)				.99
Male	29 (30)	9 (30)	21 (29)	
Female	68 (70)	21 (70)	52 (71)	
Age (years), mean (SD)	44 (17)	38 (19)	47 (18)	.048^b^
Education level, n (%)				<.001^b^
Elementary school	0 (0)	2 (7)	6 (8)	
Junior high school	2 (2)	2 (7)	8 (11)	
Senior high school	12 (12)	6 (20)	29 (40)	
College degree or higher	83 (86)	20 (67)	30 (41)	
Occupation, n (%)				.01^b^
Yes	64 (66)	13 (43)	33 (45)	
No	33 (34)	17 (57)	40 (55)	
Marriage, n (%)				.001^b^
Unmarried	36 (37)	20 (67)	27 (37)	
Married	61 (63)	9 (30)	39 (53)	
Divorced	0 (0)	1 (3)	7 (10)	

a HAMD-17: Hamilton Depression Rating Scale-17.

b Statistical significance.

**Table 3.** Demographic data of patients in the 2 suicide risk groups.

Variable	SAD PERSONS≤3 group (n=110)	SAD PERSONS≥4 group (n=90)	*P* value
Sex, n (%)			.09
Male	27 (24.5)	32 (35.6)	
Female	83 (75.5)	58 (64.4)	
Age (years), mean (SD)	45 (16.7)	42 (19.3)	.26
Education level, n (%)			<.001^a^
Elementary school	0 (0)	8 (8.9)	
Junior high school	4 (3.6)	8 (8.9)	
Senior high school	17 (15.5)	30 (33.3)	
College degree or higher	89 (80.9)	44 (48.9)	
Occupation, n (%)			.02^a^
Yes	69 (62.7)	41 (45.6)	
No	41 (37.3)	49 (54.4)	
Marriage, n (%)			<.001^a^
Unmarried	34 (30.9)	49 (54.4)	
Married	75 (68.2)	34 (37.8)	
Divorced	1 (0.9)	7 (7.8)	

a Statistical significance.

### Proposed Framework

**Figure 1.**
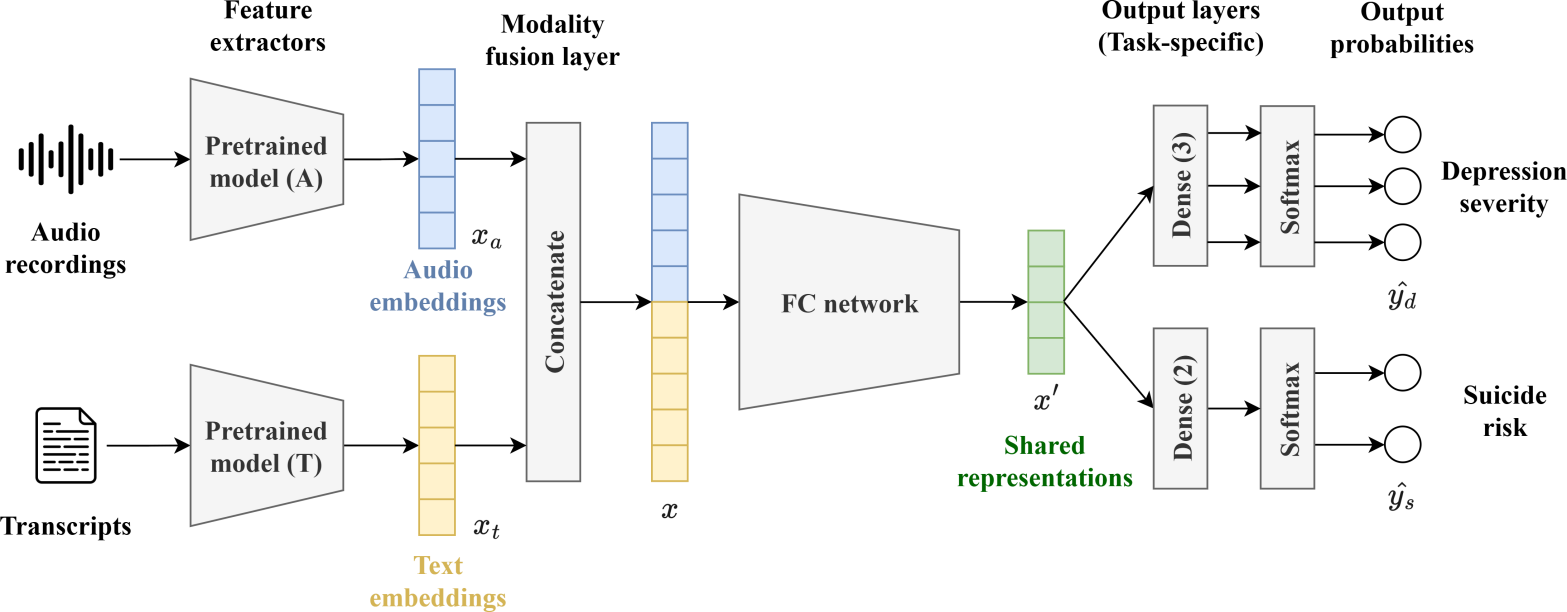
Overview of our proposed framework. FC: fully connected.

The framework comprises 3 components: feature extraction, multimodal fusion, and MTL architecture, which are discussed sequentially in the following sections (Figure 1). First, audio and text data undergo processing by pretrained models to extract their embeddings. Second, the embeddings obtained from the previous step are fused using a modality fusion layer. The resulting fused representations are then fed into a fully connected (FC) network to project them into lower-dimensional vectors. Lastly, these representations are shared between the 2 classification tasks (DS and SR) and are input into 2 task-specific layers implemented as multilayer perceptron classifiers to generate output probabilities separately. The details of the 3 components are presented below.

First, considering the small sample size in our study and recognizing the potential of TL in predicting DS and SR, we used 4 advanced pretrained models for feature extraction: wav2vec 2.0 and HuBERT for audio analysis [49,50], and Longformer and ERNIE-health for text analysis [51,52]. Each method has distinct advantages, rendering them especially suitable for our research objectives, as elaborated upon in the following sections.

- wav2vec 2.0: It is developed by Facebook AI Research, uses a multilayer convolutional neural network (CNN) for audio encoding, and is supplemented by latent representation masking and contextualization through a Transformer network trained with contrastive learning methods [49]. This self-supervised model excels with minimal labeled data, consistently surpassing state-of-the-art models, as demonstrated in the tasks of depression detection [52] and emotion recognition [53].

- HuBERT: It extends self-supervised learning to audio data, using a CNN for encoding and a BERT encoder for contextualization, enhanced by masked prediction and cluster refinement [50]. HuBERT has demonstrated superior performance in audio classification tasks for detecting depression [54] and assessing cognitive function [55].

- Longformer: It stands out as a transformer-based language model designed to capture extended dependencies using sliding window and global attention mechanisms [51]. This design enables Longformer to effectively integrate local and global information while mitigating challenges associated with traditional attention mechanisms.

- ERNIE-health: It is a Chinese biomedical language model tailored for biomedical text processing, enhancing tokenization and comprehension of biomedical content through in-domain text [56]. ERNIE-health consistently outperforms other models across various biomedical tasks [57], underscoring its effectiveness in this domain.

Second, these pretrained models were used to generate audio embeddings, $x_{a}$, and text embeddings, $x_{t}$, by feeding the preprocessed audio recordings and transcripts as their inputs. To combine the information from text and audio modalities, we adopted the early fusion approach by concatenating the audio embedding ($x_{a}$) and text embedding ($x_{t}$) into a single vector (x), using Eq. (1). This fusion strategy, also known as feature-level fusion, is characterized by its simplicity, its computational efficiency, and the potential to capture intricate interactive details. We adopted this approach due to its aforementioned advantages and its ability to circumvent the risk of information overlap or cancellation inherent in more complex operations such as addition, deduction, inner product, and outer product. This approach has been widely used in prior studies on audio-text fusion, consistently yielding improved accuracy [58-60].


(1) $$x=x_{a}⊕x_{t}$$

Lastly, our proposed framework adopted the hard parameter sharing scheme for MTL of DS and SR classification using deep learning. This scheme involves a shared encoder with multiple task-specific decoding heads [46]. This MTL technique enables our framework to learn multiple related tasks simultaneously while improving the generalization performance. In our framework, an FC network acts as the shared encoder, and dense layers act as the task-specific heads. The FC network f learns a condensed representation $x^{`}$ from the fused input x, as shown in Eq. (2). Subsequently, a softmax function is applied to 2 task-specific dense layers, $g_{d}$ and $g_{s}$, to transform $x^{`}$ into output probabilities for DS classification (Eq. (3)) and SR classification (Eq. (4)), respectively.


(2) $$x^{`}=f\left(x\right)$$

(3) $${\hat{y}}_{d}=Softmax\left(g_{d}\left(x^{\prime }\right)\right)$$

(4) $${\hat{y}}_{s}=Softmax\left(g_{s}\left(x^{\prime }\right)\right)$$

In the context of an MTL model, the design of loss functions for multiple objectives is crucial. Instead of using weighted sum of loss functions, which can be influenced by weights and time-consuming to determine, we adopted the automatic weighted loss approach introduced by [47]. This method considers the homoscedastic uncertainty of each task and derives appropriate weights based on task uncertainties. Tasks with higher uncertainties are assigned lower weights, allowing the model to effectively learn across tasks in a more balanced manner.

For the loss calculation, our proposed method involves a 2-stage approach. In the first stage, we compute task-specific losses, $L_{d}$ and $L_{s}$, for DS and SR tasks, respectively, using cross entropy, as described in Eq. (5) (C represents the number of labels in the corresponding task). In the second stage, the total loss, $L_{total}$, is determined using the automatic weighted loss method proposed by [47], as depicted in Eq. (6). The goal is to minimize the total loss, enhancing the model’s performance in DS and SR classification tasks, which can enable effective learning from the data and accurate predictions for both tasks.


(5) $$L=-\sum_{i=1}^{C}y_{i}\cdot \log({\hat{y}}_{i})$$

(6) $$L_{total}=\frac{1}{2{\sigma_{d}}^{2}}L_{d}+\frac{1}{2{\sigma_{s}}^{2}}L_{s}+\log\left({\sigma_{d}}^{2}\right)+log({\sigma_{s}}^{2})$$

### Implementation Details

We implemented our approach using PyTorch [61] and the Transformers library from Hugging Face [62]. Pretrained models were loaded by specifying the model version string in the application programming interface. Refer to Table S1 in Multimedia Appendix 1 for details of the Chinese versions of the 4 models selected for this study.

To extract features from the audio and text modalities, we configured several parameters. The audio features were generated with a sampling rate of 16,000 and a duration of 6.25 seconds, resulting in a 100,000-dimensional feature. For the text modality, transcripts were tokenized into a fixed length of 512 tokens, with truncation or padding applied if necessary. The audio features were then transformed into 1024-dimensional embeddings, while the tokenized text inputs were represented as 768-dimensional embeddings.

To prevent overfitting during training, batch normalization and rectified linear unit activation were applied to linear layers that did not act as classifiers. A batch size of 8 was used, and the models were trained for 20 epochs with an early stopping patience of 3. Cross-entropy was used to calculate the loss for single-task learning (STL), while automatic weighted loss was used for MTL. The AdamW optimizer was used for optimizing the losses. The parameter details for each model, including the modality used (single or multiple) and the learning architecture adopted (single task or multitask), are presented in Table 4.

**Table 4.** Parameter settings.

Parameter	SMSTL^a^	MMSTL^b^	SMMTL^c^	MMMTL^d^
Epochs	20	20	20	20
Early stopping patience	3	3	3	3
Batch size	8	8	8	8
Learning rate	0.0005	0.0005	0.0005	0.0005
Warmup ratio	0.3	0.35	0.35	0.25
Dropout probability	0.2	0.1	0.1	0.1

a SMSTL: single modality with single-task learning.

b MMSTL: multimodal with single-task learning.

c SMMTL: single modality with multitask learning.

d MMMTL: multimodal with multitask learning.

### Experimental Evaluation

**Figure 2.**
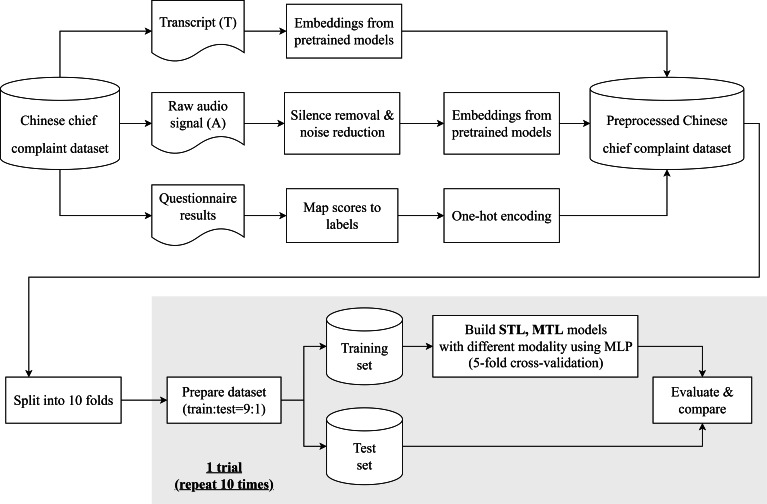
Flow diagram. MLP: multilayer perceptron; MTL: multitask learning; STL: single-task learning.

Our proposed framework is built using the 3 data types in the dataset: audio recordings, transcripts, and questionnaire results, as shown in the flow diagram in Figure 2.

During preprocessing, the audio data underwent 3 steps: removal of file-edge silence, denoising using Podcastle [63], and feature extraction. We used Podcastle’s Magic Dust AI technology for its advanced denoising capabilities, which integrate spectral filtering, adaptive noise cancellation, and machine learning algorithms [64]. Specifically, we used the “noise reduction” mode to automatically detect and suppress nonstationary background noises, such as coughs, sniffles, and microphone taps, while preserving speech clarity and signal integrity [65]. This step minimized noise-related distortions prior to feature extraction and analysis.

Feature extraction was then applied to both audio and text data using pretrained models, yielding their respective embeddings as described earlier. The processed dataset was partitioned into 10 subsets for cross-validation, with 1 subset used for testing and the remaining 9 for training in each fold. Final performance metrics were averaged across all 10 trials. In parallel, questionnaire responses were one-hot encoded to represent discrete class labels, serving as the output variables for prediction.

Our study consisted of 3 experiments. In experiments 1 and 2, we built STL models for DS and SR tasks, using different combinations of embeddings. The primary aim was to identify the best pretrained models for extracting text and audio embeddings in each task and assess the advantages of using multimodal data compared to unimodal data for each task. In experiment 3, we developed several MTL models with hard parameter sharing to combine information from both tasks. The performance of these MTL models was then compared to the STL models from experiments 1 and 2, providing insights into the potential benefits of MTL for the 2 tasks.

### Performance Measure

To assess the effectiveness of our classification models, we used a range of standard metrics, including accuracy, recall, precision, specificity, *F*_1_-score, and area under the curve (AUC). These metrics were derived from the confusion matrix, with AUC serving as the primary metric for comprehensive performance evaluation. In cases where the difference in AUC between models was not significant, we also considered other metrics, such as accuracy, *F*_1_-score, and recall, to ensure a thorough assessment of model performance.

In the SR prediction task, the positive class (eg, “at risk”) encompassed individuals with a moderate or high risk of suicide, as detailed earlier. In contrast, for the DS prediction task, the models’ performance across all classes (eg, none, low/moderate, and high) was evaluated using the macro-average approach, rather than focusing solely on a specific positive class.

## Results

### Experiment 1: STL Models for DS Prediction

**Figure 3.**
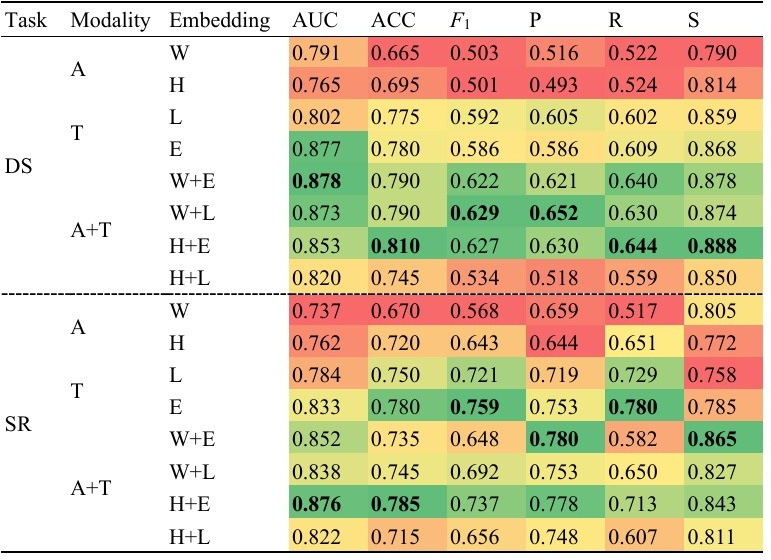
Performance comparison of single-task learning models for depression severity (DS) and suicide risk (SR) prediction. Performance metrics are presented as a heatmap, where color gradients reflect the relative magnitude of values, ranging from red (lower values) to green (higher values). A: audio only; A+T: combined audio and text; ACC: accuracy; AUC: area under the curve; E: ERNIE-health; *F*_1_: *F*_1_-score; H: HuBERT; L: Longformer; P: precision; R: recall; S: specificity; T: text only; W: wav2vec 2.0.

In experiment 1, we aimed to find the best STL model for DS classification by using various pretrained embeddings to differentiate between the 3 severity levels. These models employed a multilayer perceptron classifier for classification and were categorized into audio-only, text-only, and combined audio and text modalities based on the embeddings used. The classification performance of these DS prediction models on each metric is presented in Figure 3.

Regarding DS prediction models, we obtained several findings (Figure 3). First, the results demonstrated that most embeddings, except those of the audio modality, performed well in terms of AUC, with scores exceeding 0.8. In the audio modality, the wav2vec 2.0 embedding outperformed the HuBERT embedding. In the text modality, the ERNIE-health embedding demonstrated superior performance in terms of AUC (0.877), accuracy (0.780), recall (0.609), and specificity (0.868), indicating its effectiveness in capturing specific aspects of DS in textual data. Second, combining embeddings from different modalities led to improvements across all metrics for most embeddings. Notably, the addition of the ERNIE-health embedding to the HuBERT embedding resulted in a substantial performance boost, with an 11.5% increase in AUC and up to 27.79% improvement in precision. Third, our comprehensive evaluation of multiple metrics showed that the multimodal models outperformed the single-modality models, except for the combination that included the HuBERT embedding, which may impair the ability of text embeddings. Lastly, among all the embeddings analyzed, the wav2vec 2.0+ERNIE-health and wav2vec 2.0+Longformer embeddings achieved the highest AUC scores of 0.878 and 0.873, respectively.

### Experiment 2: STL Models for SR Prediction

In experiment 2, our objective was to identify the best STL model for SR classification by using different pretrained embeddings. Similar to experiment 1, multilayer perceptron classifiers were used to analyze the embeddings from different modalities. The evaluation results of these embeddings for SR classification are presented in Figure 3.

Based on the data presented in Figure 3, regarding SR prediction models, several findings were obtained. First, the results demonstrated that most embeddings achieved AUC values greater than 0.8, except for audio modality embeddings and the Longformer embedding. In the audio modality, the HuBERT embedding outperformed the wav2vec 2.0 embedding on most metrics, except for precision and specificity. This suggests that the HuBERT embedding may be a better choice for overall SR classification, while the wav2vec 2.0 embedding may be more effective in correctly identifying individuals who are not at risk of suicide. In the text modality, the ERNIE-health embedding outperformed the Longformer embedding, obtaining higher values on all metrics, indicating that the ERNIE-health embedding is more effective for SR classification. Second, combining embeddings from different modalities consistently improved AUC, precision, and specificity. Specifically, incorporating multimodal embeddings led to significant performance improvements, with increased AUC (2.28% to 15.60%), precision (3.32% to 20.81%), and specificity (2.73% to 10.19%) across all single-modality models, indicating improved accuracy in identifying nonrisk individuals. Third, the HuBERT+ERNIE-health embedding achieved the highest performance in terms of AUC (0.876) among all embeddings.

### Experiment 3: MTL Models for DS and SR Predictions

**Figure 4.**
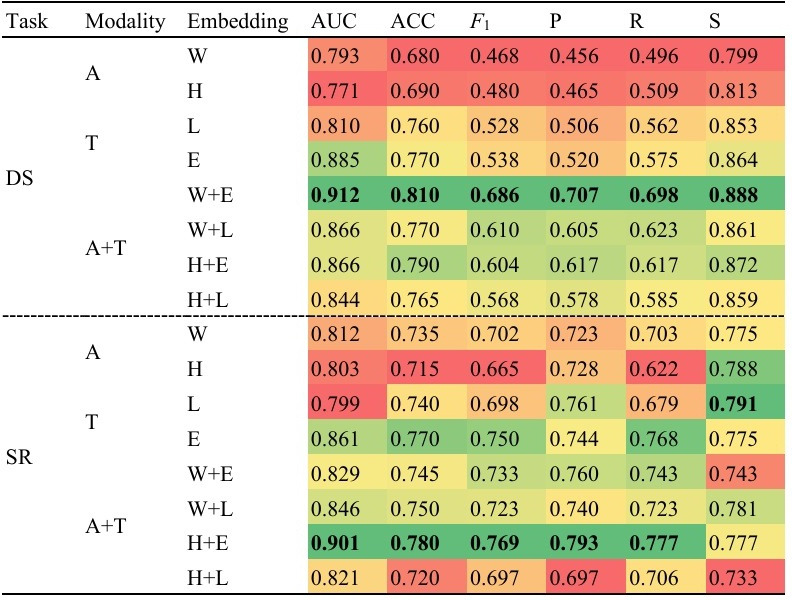
Performance comparison of multitask learning models for depression severity (DS) and suicide risk (SR) prediction. Performance metrics are presented as a heatmap, where color gradients reflect the relative magnitude of values, ranging from red (lower values) to green (higher values). A: audio only; A+T: combined audio and text; ACC: accuracy; AUC: area under the curve; E: ERNIE-health; *F*_1_: *F*_1_-score; H: HuBERT; L: Longformer; P: precision; R: recall; S: specificity; T: text only; W: wav2vec 2.0.

In experiment 3, we aimed to explore the potential of MTL models in improving DS and SR predictions by leveraging shared information between the 2 tasks. Figure 4 provides a comprehensive summary of the performance metrics, and the subsequent content further discusses the results of experiments 1 and 2 for comparison.

From Figures3  4, we found that all models, except the ones using the wav2vec 2.0+Longformer embedding, demonstrated an increase in AUC ranging from 0.25% to 3.88% with MTL, indicating the potential of MTL in enhancing performance for DS. Additionally, we observed that when adopting MTL for SR prediction, all models, except for the wav2vec 2.0+ERNIE-health and HuBERT+Longformer embeddings, demonstrated an increase in AUC ranging from 0.96% to 10.18%. On the other hand, what stands out is that when applying the MTL framework, there was a consistent enhancement in accuracy, *F*_1_-score, and recall among the combined audio and text models, including the aforementioned 2 models using the wav2vec 2.0+ERNIE-health and HuBERT+Longformer embeddings. These findings suggest that combined audio and text embeddings are well-suited for the MTL approach, although they may increase false positives while better identifying individuals at risk for suicide.

## Discussion

### Principal Findings

This study proposes a multitask framework that integrates a multimodal fusion strategy using pretrained audio and text embeddings to concurrently assess DS and SR. The efficacy of the proposed method has been validated using real-world clinical data.

Some of the significant findings of this study are as follows. First, we introduced and investigated renowned pretrained models for their effectiveness in audio and text classification tasks. The findings demonstrated that the ERNIE-health text modality embedding, specifically trained on a medical corpus, consistently outperformed the Longformer text modality embedding in both STL models (for DS prediction and SR prediction) and MTL models. On the other hand, the wav2vec 2.0 audio modality embedding performed better than the HuBERT embedding in STL models for DS prediction and MTL models for both tasks, but performed worse than the HuBERT embedding in STL models for SR prediction.

Second, our results underscore the effectiveness of multimodal approaches over single-modality ones in classifying DS and SR in the majority of cases. Even straightforward fusion techniques, such as concatenation, improve performance by integrating richer information, consistent with previous research [30,31,66]. This implies that the combination of audio and text embeddings provides a more comprehensive representation of the underlying phenomena than using each modality independently.

Third, the results indicated that the performance of text modality models significantly surpassed that of audio modality models, except in MTL models using the Longformer embedding for SR prediction. Several potential explanations can be considered for this observation. Despite preprocessing efforts to reduce noise, the audio modality model remains susceptible to variations in speaker accents or weaker emotional expressiveness [67], which can adversely affect the model’s performance. In contrast, text data are not influenced by such variations. Additionally, techniques for processing and embedding text data are highly advanced, such as ERNIE-health, which can contribute to the superior performance of most text modality models. This demonstrates that ERNIE-health can effectively bridge the gap between pretraining goals and downstream tasks [56]. Conversely, processing and feature extraction for audio data in our dataset may not be as efficient as for text embeddings. Furthermore, research indicates that suicidal tendencies and depressive symptoms are explicitly conveyed through syntactic and semantic patterns in text, which are efficiently captured by text embeddings [68]. On the contrary, extracting and interpreting these signals from audio data are inherently more complex and less robust.

Fourth, our findings demonstrated that the proposed MTL framework, using specific pretrained audio and text embeddings, significantly enhanced the classification performance for DS and SR. Considering the common class imbalance in clinical datasets, we selected AUC as our primary evaluation metric to provide a reliable and clinically meaningful representation of model performance [69,70]. However, our experiments also revealed that not all models benefited from its use, with 3 multimodal models showing no improvement in AUC (Table 5). Interestingly, MTL improved the performance of all single-modality models on both tasks. However, among the 4 multimodal models, only 1 (ie, using the HuBERT+ERNIE-health embeddings) exhibited improvement in AUC for both tasks when using MTL. The remaining 3 models demonstrated mixed results, with 1 task showing improvement, and the others experiencing a drop in performance. This highlights the phenomenon of negative transfer [71], suggesting that transferred knowledge may not always have a positive impact on other tasks, even if they share similarities [71].

**Table 5.** Performance comparison of STL^a^ and MTL^b^ models for depression severity and suicide risk prediction.

Task, modality, and embedding			AUC^c^		Improvement
			STL	MTL	
Depression severity prediction					
Audio only					
wav2vec			0.791	0.793	+0.002
HuBERT			0.765	0.771	+0.006
Text only					
Longformer			0.802	0.810	+0.008
ERNIE-health			0.877	0.885	+0.008
Combination of audio and text					
wav2vec+ERNIE-health			0.878	0.912^d^	+0.034
wav2vec+Longformer			0.873	0.866	−0.007
HuBERT+ERNIE-health			0.853	0.866	+0.013
HuBERT+Longformer			0.820	0.844	+0.024
Suicide risk prediction					
Audio only					
wav2vec			0.737	0.812	+0.075
HuBERT			0.762	0.803	+0.041
Text only					
Longformer			0.784	0.799	+0.015
ERNIE-health			0.833	0.861	+0.028
Combination of audio and text					
wav2vec+ERNIE-health			0.852	0.829	−0.023
wav2vec+Longformer			0.838	0.846	+0.008
HuBERT+ERNIE-health			0.876	0.901^d^	+0.025
HuBERT+Longformer			0.822	0.821	−0.001

a STL: single-task learning.

b MTL: multitask learning.

c AUC: area under the curve.

d Highest AUC values for each task.

Finally, our study revealed that MTL models led to more substantial improvements in the SR prediction task compared to the DS prediction task, with all multimodal MTL models demonstrating higher recall than their STL counterparts in predicting SR. These findings may be attributed to several aspects. On one hand, the SR prediction task may involve information or patterns different from those in the DS prediction task. For instance, text modalities might convey clearer linguistic patterns, such as specific word choices, pronoun usage, and negative terms [17], which could be more predictive of SR than DS. However, MTL allows models to share learned representations across tasks. If the features relevant to the SR prediction task benefit from certain text or audio modality representations, these features may also aid the DS task, even if the latter shows less improvement. On the other hand, the prediction of DS may be more influenced by sample variability [72,73], whereas the prediction of SR might exhibit stronger commonalities across samples. These findings further underscore the value of MTL, as it enables the model to address such differences through shared representations, thereby enhancing prediction accuracy.

To contextualize our work within current state-of-the-art techniques, we compared our multitask framework with recent studies on depression and suicide prediction, as summarized in Table S2 in Multimedia Appendix 1. Our proposed MTL model, which integrates audio and text modalities with pretrained embeddings, achieved competitive performance (DS: AUC=0.91; accuracy=0.81; *F*_1_-score=0.69 with wav2vec 2.0+ERNIE-health; SR: AUC=0.90; accuracy=0.78; *F*_1_-score=0.77 with HuBERT+ERNIE-health), outperforming several prominent MTL models. These include models by Benton et al [25] (depression: AUC=0.77; suicide: AUC=0.83), Ghosh et al [46] (depression: accuracy=0.74), and Yang et al [27] (suicide: accuracy=0.74). While Buddhitha and Inkpen [29] reported slightly higher performance for suicide prediction (AUC=0.88; accuracy=0.84), their approach relied on Reddit posts rather than clinical data.

Our study also outperformed all single-task depression prediction studies presented in Table S2 in Multimedia Appendix 1, which predominantly used binary classification (ie, depressed vs nondepressed). In contrast, our multitask framework enabled a more nuanced assessment by explicitly predicting the severity of depressive symptoms rather than merely classifying their presence or absence. Although some single-task suicide prediction models reported higher metrics, including models by Chen et al [38] (*F*_1_-score=0.76), Tsui et al [39] (AUC=0.93), and Bouktif et al [36] (accuracy=0.94), they used substantially larger datasets (1284 subjects, 45,238 patients, and 3,48,110 posts, respectively) and focused exclusively on single-task prediction. Similarly, Ramírez-Cifuentes et al [40] achieved an AUC of 0.94 for suicide prediction using social media data, which suffered from known limitations, including self-presentation biases, language ambiguities, and an inability to detect offline SR [30].

Our study uniquely applied MTL to simultaneously predict DS and SR using multimodal data from clinical interviews. Unlike prior work that focused on single tasks or unimodal inputs, often derived from electronic health records or social media, our approach captured direct clinical interactions, yielding more authentic behavioral signals. Comparative analyses demonstrated that our model effectively predicted both DS and SR, offering clear advantages over existing methods for this clinically important objective.

### Theoretical Implications

This study makes substantial contributions to existing literature from 2 main perspectives. First, this study delineated the efficacy of integrating MML, MTL, and TL in simultaneously identifying DS and SR, thereby advancing the understanding of depression and suicide detection. While existing research, such as [25], has explored the impact and importance of MTL in DS and SR prediction, studies have predominantly focused on social media contexts. Limited research has evaluated the effectiveness of MTL in clinical settings. This study addressed this gap through empirical experiments using real-world clinical datasets, demonstrating that the proposed multimodal multitask approach, integrating pretrained embeddings, is applicable to clinical settings.

Furthermore, our findings underscore that MTL generally enhances model performance, consistent with prior literature (eg, [25,45,46]), highlighting the benefits of knowledge sharing across domains [25]. However, our experiments also revealed instances of negative transfer [71], emphasizing the importance of selecting optimal MTL strategies based on embeddings, tasks, and application scenarios. Moreover, further thoughtful evaluation should consider balancing the costs associated with false positives and false negatives, using more comprehensive metrics.

Second, we discussed and presented a comparison of popular pretrained models (Longformer and ERNIE-health for text modality, and wav2vec 2.0 and HuBERT for audio modality) to evaluate their effectiveness with clinical data, providing a valuable addition to the existing literature on depression and suicide prediction research. Our findings revealed that ERNIE-health outperformed Longformer in text modality embedding, and wav2vec 2.0 generally surpassed HuBERT, although there were instances where the reverse was true. This underscores the necessity of judicious pretrained model selection and thorough testing for clinical applicability in the future. Nevertheless, we still affirm the efficacy of TL, as even single-task and single-modality models exhibited commendable performance, although our dataset included only 200 samples.

### Practical Implications

This study has several important practical implications. First, the persistent challenge of data scarcity has limited progress in both academic research and clinical practice. Through techniques like MML, TL, and MTL, we propose promising solutions. Second, by integrating multimodal data from speech and text and applying TL methods, our approach can facilitate clinical diagnosis with objective and quantitative measurements. This enables a rapid, efficient, and cost-effective assessment of DS and SR based solely on patients’ verbal disclosures to health care providers. Third, the effectiveness of our method suggests a promising avenue for automated SR detection through the development of innovative tools, thereby making a significant contribution to early suicide prevention efforts.

### Limitations and Future Research

This study has certain limitations that warrant further research. First, our dataset of 200 participants (100 patients with depression and 100 healthy individuals) represents a significant limitation that severely constrains the generalizability of our findings to broader populations. Despite implementing cross-validation techniques, this small sample size introduces considerable risks of overfitting, where the model may capture dataset-specific characteristics rather than robust, generalizable patterns for DS and SR detection. This limitation necessitates external validation with larger, more diverse cohorts from different clinical settings and demographic backgrounds to establish the true clinical utility and robustness of our proposed method. The incorporation of larger external datasets is therefore essential to not only enhance robustness but also refine and validate our approach across varied populations.

Furthermore, addressing data imbalance has emerged as a critical challenge in accurately identifying and classifying depression cases across varying severity levels. Our comprehensive analysis revealed significant performance disparities among “none,” “low/moderate,” and “high” severity subcategories, with particularly pronounced difficulties in classifying “low/moderate” severity cases (Table S3 in Multimedia Appendix 1). This variability underscores the intricate complexity of developing a robust diagnostic approach capable of consistently discerning nuanced variations in DS. Future research should, therefore, focus on advancing MTL strategies that integrate multimodal feature representations with targeted sampling techniques and refined weighting mechanisms to enhance the robustness of model predictive performance across varying severity levels of depression.

Furthermore, the exploration of diverse fusion strategies and weight adjustments in MTL, along with the investigation of various pretrained models, warrants further investigation to potentially enhance model performance in future studies. However, while our implementation was straightforward, our primary objective was to develop a computationally efficient and effective method that prioritizes resource efficiency. Finally, exploring the applicability of these techniques to a broader spectrum of mental health disorders is essential. This includes leveraging MML and MTL approaches to integrate information across different disorders, thereby expanding the scope of potential applications in mental health diagnostics.

### Conclusion

Early detection and accurate diagnosis are crucial for implementing timely interventions and alleviating the societal and economic burdens associated with mental health conditions. This study proposes an effective approach to improving model performance by integrating MTL, MML, and TL for concurrent depression and suicide detection. Our empirical findings, obtained by fine-tuning MTL models on clinical datasets, provide compelling evidence for the effectiveness of integrating MTL, MML, and TL methods in addressing mental health tasks. However, we advocate for cautious MTL implementation to mitigate potential negative transfer effects. Additionally, we recommend careful consideration for the selection of pretrained models and rigorous validation to ensure their clinical applicability. Our proposed methods offer a promising pathway for future research and clinical applications in mental health diagnostics.

## Supplementary material

10.2196/66907 Multimedia Appendix 1 Additional data to support the findings of the study.

## Abbreviations

AUC: area under the curve
CNN: convolutional neural network
DS: depression severity
FC: fully connected
HAMD-17: Hamilton Depression Rating Scale-17
MML: multimodal learning
MTL: multitask learning
SR: suicide risk
STL: single-task learning
TL: transfer learning
