# Supplementary material for: Multimodal Multitask Learning for Predicting Depression Severity and Suicide Risk Using Pretrained Audio and Text Embeddings: Methodology Development and Application
Source: JMIR Med Inform. 2025 Oct 30;13:e66907. doi: 10.2196/66907 (PMC12574750; doi:10.2196/66907)
Supplement: Multimedia Appendix 1 [file medinform-v13-e66907-s001.docx]

**Multimedia Appendix**

**Table S1**: Versions of pretrained models.

| Modality | Model | Pretrained version |
| --- | --- | --- |
| Audio | wav2vec 2.0 | ydshieh/wav2vec2-large-xlsr-53-chinese-zh-cn-gpt |
|  | HuBERT | TencentGameMate/chinese-hubert-large |
| Text | Longformer | ValkyriaLenneth/longformer_zh |
|  | ERNIE-health | nghuyong/ernie-health-zh |

**Table S2**: Comparative analysis of our proposed MTL framework against state-of-the-art models for depression and suicide prediction.

| **Study** | **Tasks** | **Modalities** | **Dataset (Samples)** | **Methods** | **Performance** |
| --- | --- | --- | --- | --- | --- |
| Our Study | DS & SR (MTL) | Text & Audio | Clinical interviews (100 patients and 100 healthy controls) | DNN with MTL and pretrained embeddings | DS: AUC=0.91, ACC=0.81, F1=0.69;  SR: AUC=0.90, ACC=0.78, F1=0.77 |
| [1] | Depression & Suicide (MTL) | Text | Twitter dataset (9,611 users) | DNN with MTL | Depression: AUC=0.77;  Suicide: AUC=0.83 |
| [2] | Depression (MTL) | Text | CEASE (2,539 sentences) | Bi-GRU | ACC=0.74 |
| [3] | Suicide (MTL) | Text | CEASE (2,393 sentences) | BERT | ACC=0.74 |
| [4] | Suicide (MTL) | Text | CLPsych 2019, UMD, SMHD (309,670 users) | CNN | Suicide w/ depression: AUC=0.88, ACC=0.84, F1=0.78 |
| [5] | Depression | Text & Audio | DAIC-WOZ, CMU-MOSEI (189 recordings) | LSTM | ACC=0.64, F1=0.60 |
| [6] | Depression | Text & Audio | DAIC-WOZ (189 recordings) | TOAT transformer-based model | ACC=0.74, F1=0.65 |
| [7] | Depression | Text & Audio | DAIC-WOZ & EDAIC-WOZ (180 recordings) | BiLSTM | AUC=0.73 |
| [8] | Depression | Text | EHR database (10,148 patients) | Lasso, SVM, MLP, RF | AUC=0.81 |
| [9] | Depression | Text | CLEF2017 (887 posts) | CNN-BiLSTM | AUC=0.85 |
| [10] | Depression | Audio | DAIC-WOZ (189 recordings) | LSTM | ACC=0.76, F1=0.67 |
| [11] | Depression | Audio | Smartphone (318 recordings) | CNN | ACC=0.78 |
| [12] | Suicide | Text & Images | Twitter dataset (252 users) | LR | AUC=0.94 |
| [13] | Suicide | Text & Audio | Crisis hotlines (1,284 recordings) | LSTM, BiLSTM, GNN, Transformer | F1=0.76 |
| [14] | Suicide | Text & EHR | UPMC Medical System (45,238 patients) | NB, Lasso, RF, EXGB | AUC=0.93 |
| [15] | Suicide | Text | Reddit posts (348,110 posts) | CNN, LSTM, BERT | ACC=0.94 |
| [16] | Suicide | Text | Facebook posts (83,292 posts) | ANN | AUC=0.70-0.75 |
| [17] | Suicide | Audio | Crisis hotlines (164 recordings) | LR, SVM, RF, XGB | ACC=0.75, F1=0.70 |
| Note: MTL=multitask learning, DS=depression severity, SR=suicide risk, DNN=deep neural network, LSTM=long short-term memory, TOAT=topic attentive, Bi-GRU=bidirectional gated recurrent unit, BiLSTM=bidirectional long short-term memory, CNN=convolutional neural network, ANN=artificial neural network, BERT=Bidirectional Encoder Representations from Transformers, GNN=graph neural network, LR=logistic regression, NB=naive bayes, SVM=support vector machine, MLP=multi-layer perceptron, RF=random forest, EXGB=extreme gradient boosting, NA=not available (not provided in the literature). Dataset abbreviations: DAIC-WOZ=Distress Analysis Interview Corpus-Wizard of Oz, EDAIC-WOZ=Extended Distress Analysis Interview Corpus-Wizard of Oz, CMU-MOSEI=CMU Multimodal Opinion Sentiment and Emotion Intensity, CEASE=Corpus of English Suicide notes with Emotion Annotations, UMD=University of Maryland Reddit Suicidality Dataset, SMHD=Self-reported Mental Health Diagnoses dataset, EHR=electronic health records, UPMC=University of Pittsburgh Medical Center, AUC=area under the curve, ACC=accuracy, F1=F1-score. | | | | | |

**Table S3**: Performance evaluation of proposed MTL framework across depression severity subcategories: detailed metrics by embedding approach and severity level.

| **Embedding** | **Severity Level** | **AUC** | **ACC** | **F1** | **P** | **R** | **S** |
| --- | --- | --- | --- | --- | --- | --- | --- |
| W+E | None | 0.926 | 0.888 | 0.863 | 0.854 | 0.888 | 0.808 |
|  | Low/moderate | 0.886 | 0.383 | 0.387 | 0.450 | 0.383 | 0.972 |
|  | High | 0.924 | 0.821 | 0.807 | 0.816 | 0.821 | 0.884 |
| H+E | None | 0.936 | 0.896 | 0.861 | 0.841 | 0.896 | 0.786 |
|  | Low/moderate | 0.750 | 0.150 | 0.183 | 0.250 | 0.150 | 0.989 |
|  | High | 0.912 | 0.767 | 0.804 | 0.760 | 0.804 | 0.843 |
| Note: MTL=multitask learning, AUC=area under the curve, ACC=accuracy, F1=F1 score, P=precision, R=recall, S=specificity, W=wav2vec 2.0, H=HuBERT, E=eHealth. (a) This Table S3 presents two distinctive embedding approaches selected for their optimal performance in specific diagnostic tasks. The W+E (wav2vec 2.0 and eHealth Embedding) combination was chosen for its superior effectiveness in depression severity (DS) task, while the H+E (HuBERT and eHealth Embedding) approach demonstrated optimal results for suicide risk (SR) prediction (see Table 6). (b) The performance analysis reveals important insights. A notable pattern is the substantial performance variability across different severity levels, with “Low/moderate” severity cases consistently presenting the most challenging classification scenario. In contrast, “None” and “High” severity levels exhibited more consistent performance metrics. This pronounced performance gradient, particularly evident in “Low/moderate” severity classification, underscores the intricate complexity inherent in depression severity assessment. | | | | | | | |

**References**

[1] Benton A, Mitchell M, Hovy D. Multi-task learning for mental health using social media text. arXiv preprint arXiv:1712.03538. 2017; doi: https://doi.org/10.48550/arXiv.1712.03538

[2] Ghosh S, Ekbal A, Bhattacharyya P. A multitask framework to detect depression, sentiment and multi-label emotion from suicide notes. Cognitive Computation. 2022; 14(1), 110-129. doi: https://doi.org/10.1007/s12559-021-09828-7

[3] Yang Q, Zhou J, Wei Z. Time perspective-enhanced suicidal ideation detection using multi-task learning. International Journal of Network Dynamics and Intelligence. 2024; 3(2): 100011. doi: https://doi.org/10.53941/ijndi.2024.100011

[4] Buddhitha P, Inkpen D. Multi-task learning to detect suicide ideation and mental disorders among social media users. Frontiers in Research Metrics and Analytics. 2023; 8: 1152535. doi: https://doi.org/10.3389/frma.2023.1152535

[5] Qureshi SA, Dias G, Hasanuzzaman M, Saha S. Improving depression level estimation by concurrently learning emotion intensity. IEEE Computational Intelligence Magazine. 2020; 15(3): 47-59. doi: https://doi.org/10.1109/MCI.2020.2998234

[6] Cusack CE, Ralph-Nearman C, Christian C, Fisher AJ, Levinson CA. Understanding heterogeneity, comorbidity, and variability in depression: Idiographic models and depression outcomes. Journal of Affective Disorders. 2024; 356: 248-256. doi: https://doi.org/10.1016/j.jad.2024.04.034

[7] Nykoniuk M, Basystiuk O, Shakhovska N, Melnykova N. Multimodal data fusion for depression detection approach. Computation. 2025; 13(1): 9. doi: https://doi.org/10.3390/computation13010009

[8] Melhem NM, Porta G, Oquendo MA, Zelazny J, Keilp JG, Iyengar S, Burke A, Birmaher B, Stanley B, Mann JJ. Severity and variability of depression symptoms predicting suicide attempt in high-risk individuals. JAMA Psychiatry. 2019; 76(6): 603-613. doi: https://doi.org/10.1001/jamapsychiatry.2018.4513

[9] Thekkekara JP, Yongchareon S, Liesaputra V. An attention-based CNN-BiLSTM model for depression detection on social media text. Expert Systems with Applications. 2024; 249: 123834. doi: https://doi.org/10.1016/j.eswa.2024.123834

[10] Rejaibi E, Komaty A, Meriaudeau F, Agrebi S, Othmani A. MFCC-based recurrent neural network for automatic clinical depression recognition and assessment from speech. Biomedical Signal Processing and Control. 2022; 71: 103107. doi: https://doi.org/10.48550/arXiv.1909.07208

[11] Kim AY, Jang EH, Lee S-H, Choi K-Y, Park JG, Shin H-C. Automatic depression detection using smartphone-based text-dependent speech signals: Deep convolutional neural network approach. Journal of medical Internet research. 2023; 25: e34474. doi: https://doi.org/10.2196/34474

[12] Ramírez-Cifuentes D, Freire A, Baeza-Yates R, Puntí J, Medina-Bravo P, Velazquez DA, Gonfaus JM, Gonzàlez J. Detection of suicidal ideation on social media: Multimodal, relational, and behavioral analysis. Journal of Medical Internet Research. 2020; 22(7): e17758. doi: https://doi.org/10.2196/17758

[13] Chen Y, Li J, Song C, Zhao Q, Tong Y, Fu G. Deep Learning and Large Language Models for Audio and Text Analysis in Predicting Suicidal Acts in Chinese Psychological Support Hotlines. arXiv preprint arXiv:2409.06164. 2024. doi: https://doi.org/10.48550/arXiv.2409.06164

[14] Tsui FR, Shi L, Ruiz V, Ryan ND, Biernesser C, Iyengar S, Walsh CG, Brent DA. Natural language processing and machine learning of electronic health records for prediction of first-time suicide attempts. JAMIA open. 2021; 4(1): ooab011. doi: https://doi.org/10.1093/jamiaopen/ooab011

[15] Bouktif S, Khanday AMUD, Ouni A. Explainable predictive model for suicidal ideation during COVID-19: Social media discourse study. Journal of Medical Internet Research. 2025; 27: e65434. doi: https://doi.org/10.2196/65434

[16] Ophir Y, Tikochinski R, Asterhan CS, Sisso I, Reichart R. Deep neural networks detect suicide risk from textual facebook posts. Scientific Reports. 2020; 10(1): 16685. doi: https://doi.org/10.1038/s41598-020-73917-0

[17] Su Z, Jiang H, Yang Y, Hou X, Su Y, Yang L. Acoustic features for identifying suicide risk in crisis hotline callers: Machine learning approach. Journal of Medical Internet Research. 2025; 27: e67772. doi: https://doi.org/10.2196/67772
